# Supplementary material for: Time-of-day-dependent responses of cyanobacterial cellular viability against oxidative stress
Source: Sci Rep. 2020 Nov 18;10:20029. doi: 10.1038/s41598-020-77141-8 (PMC7676254; doi:10.1038/s41598-020-77141-8)
Supplement: Supplementary file 1 — Supplementary Information. [file 41598_2020_77141_MOESM1_ESM.docx]

**Supplementary Information**

Time-of-day dependent responses of cyanobacterial cellular viability against oxidative stress

**Authors**

*Kenya Tanaka^1^, Ginga Shimakawa^2,3^ and Shuji Nakanishi^1,3,*^*

^1^ Graduate School of Engineering Science, Osaka University, 1-3 Machikaneyama, Toyonaka, Osaka 560-8631, Japan

^2^ Institute for Integrative Biology of the Cell (I2BC), CEA, CNRS, Université Paris-Sud, Université Paris-Saclay, 91198 Gif-sur-Yvette cedex, France

^3^ Research Center for Solar Energy Chemistry, Osaka University, 1-3 Machikaneyama, Toyonaka, Osaka 560-8631, Japan

**
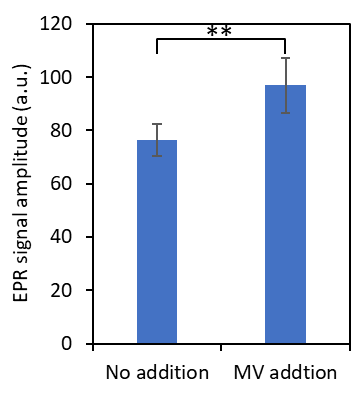
**

**Figure S1.** Effect of ROS level measured by spin trapping EPR spectroscopy on presence of 50 μM Methyl viologen (MV). The EPR signal values for different subjective times were averaged (Data is identical with Fig. 2B). Values are means ± SD of average values at seven different subjective time. Student’s t test was significant (***P* < 0.001).


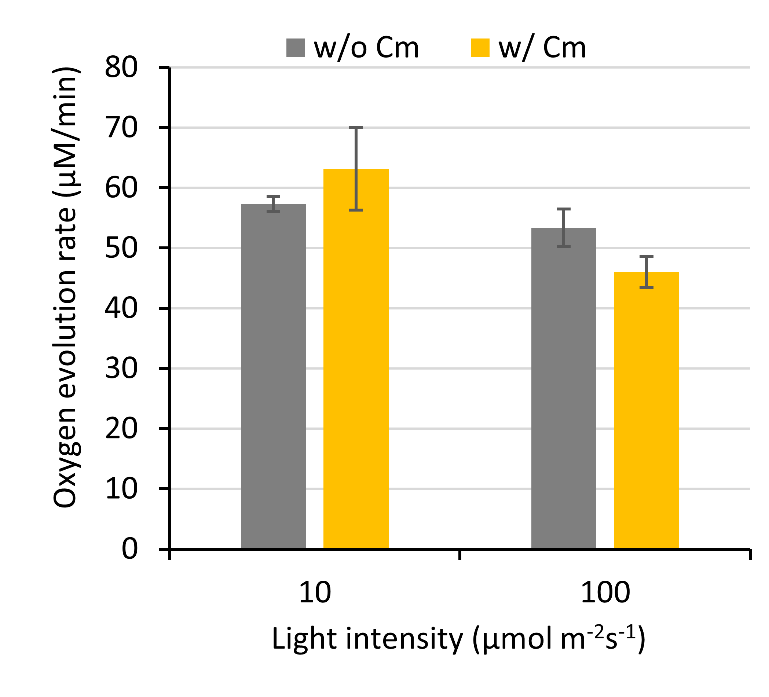


**Figure S2.** Effect of chloramphenicol (Cm) and light irradiation on PSII activity. Cells were incubated with or without 200 μg/mL Cm under light irradiation (10 or 100 µmol/m^2^/s) for 30 min. Oxygen evolution rate of reaction mixture containing BG-11 (40 mM TES-NaOH, pH 7.5), the treated cells (10 µg Chl/mL) and 0.4 mM 2,6-dichlorobenzoquinone (DCBQ) was measured under actinic light irradiation. Oxygen evolution rate of the cells incubated with Cm under 100 µmol/m^2^/s light irradiation was significantly lower than that of cells incubated without Cm (student’s t-test, *P* < 0.05), while cells incubated under 10 µmol/m^2^/s light irradiation showed no significant difference between with and without Cm. Values are means ± SD (bars) results from three independent experiments.

**A**


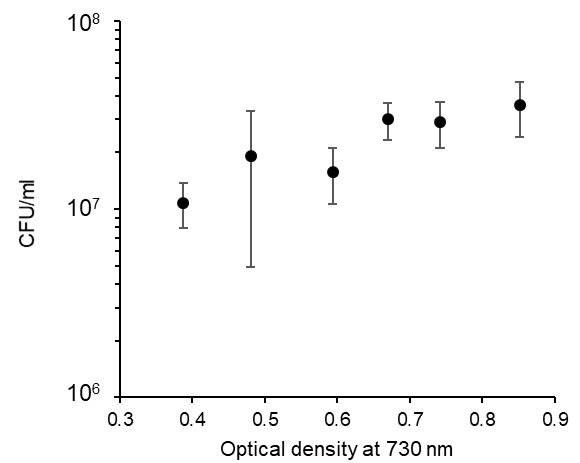


**B**


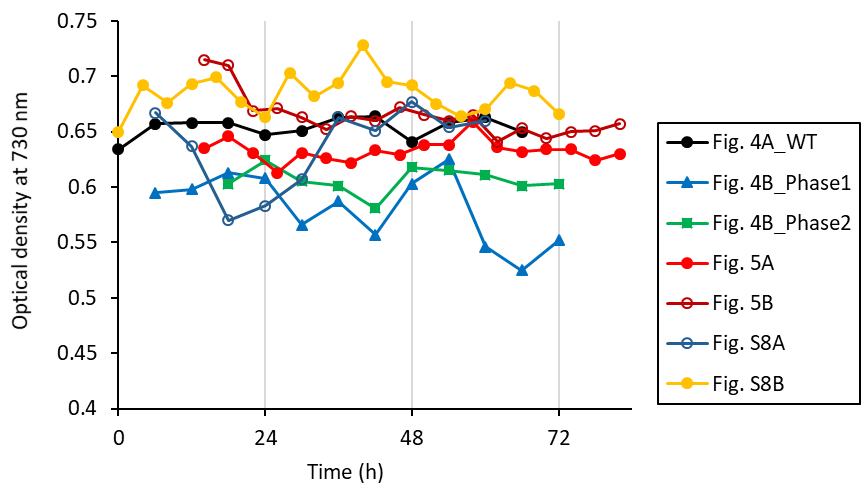


**Figure S3.** Effect of cell density during the MV/Light treatment on colony forming unit (CFU). (A) WT cell suspensions with various cell density (measured as optical density at 730 nm; OD_730_) were treated by the MV/Light treatment, followed by growing on agar plates. Values are means ± SD (bars) results from three biological replicates. (B) Time courses of OD_730_ values of cells taken for the experiments in which time-dependent ROS-tolerance change in continuous light condition was investigated (Figs. 4, 5, 6A). The time courses of the OD_730_ are hardly rhythmic, and the perturbations of the OD_730_ values are suggested not to cause significant CFU difference, indicating that the rhythmic CFU changes observed in Figs. 4, 5, 6A were not attributed to effect of time dependent cell density change.


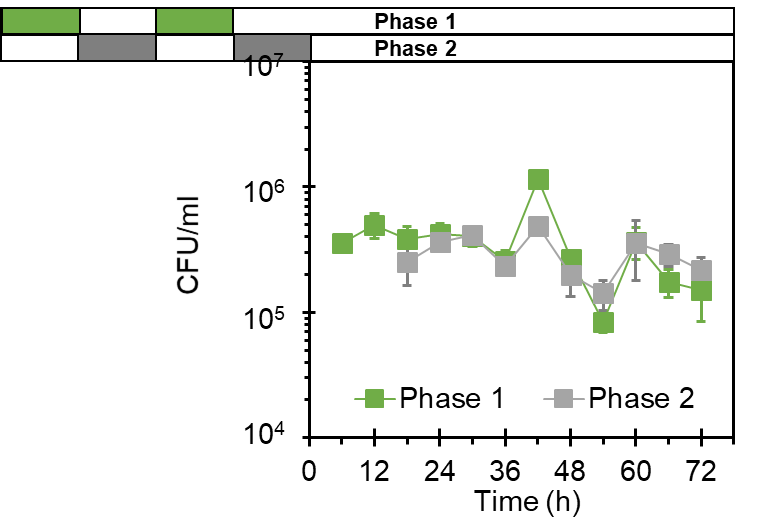


**Figure S4.** Time series of CFU of wild type cells after 40 µmol m^-2^ s^-1^-MV/light treatment. ROS tolerance of WT cell cultures entrained in antiphase were tested every 6 h. Unlike Fig. 4, time-dependent effect of circadian clock was not clearly observed when the MV/light treatments were performed at the light intensity of 40 µmol m^-2^ s^-1^ (the same intensity used for the pre-culture). White and green or gray bars denote light-dark (LD) cycles. Values and error bars represent means ± SD of three technical replicates.

**A**


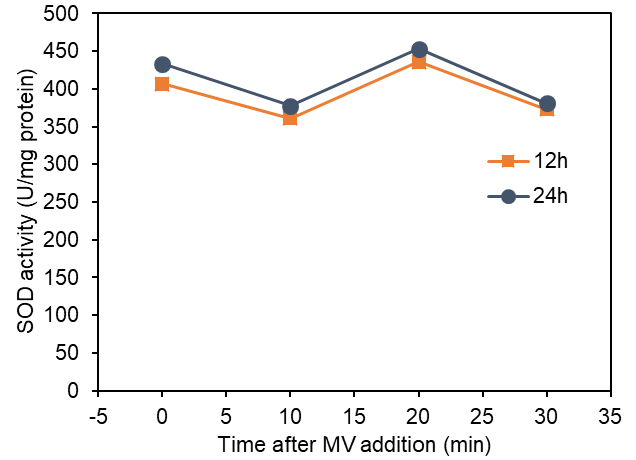


**B**


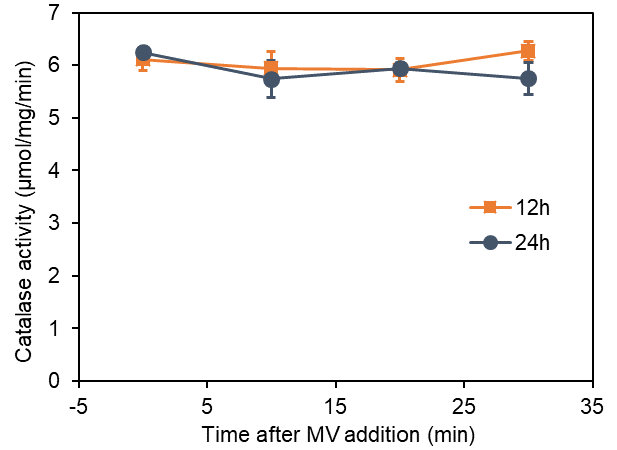


**Figure S5.** (A) Superoxide dismutase (SOD) and (B) catalase activity change during MV/light treatment (10 µmol m^-2^ s^-1^). WT cells grown in continuous light (LL) condition were picked up at 12 h or 24 h after transferring LL condition. The cells were MV/light-treated for indicated time, and cells were disrupted by mixing zirconia beads. The crude extract was collected by centrifugation at 12,000 ×g for 10 min at 4 ºC. Catalase activity was measured at 25 ºC with a Clark-type electrode in PBS (pH 7) in the presence of 1mM H2O2 as substrate using a final protein concentration of 5 µg ml^-1^. SOD activity was measured by using SDO assay kit-WST (Dojindo Kumamoto, Japan). The protein concentration of the crude extracts was determined using Qubit assay protein assay kit (Thermo Fisher).

**B**

**A**


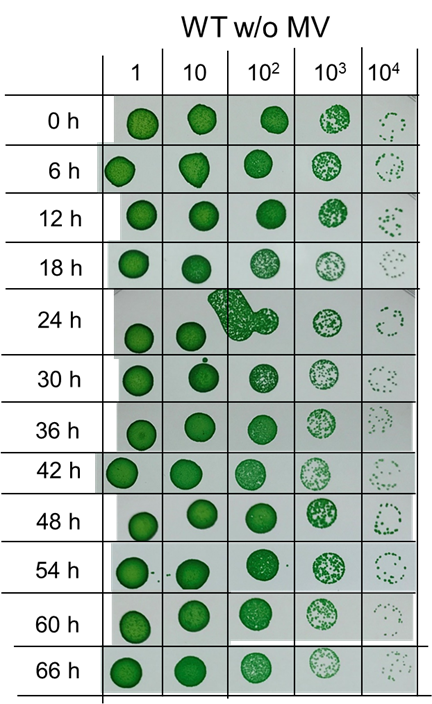

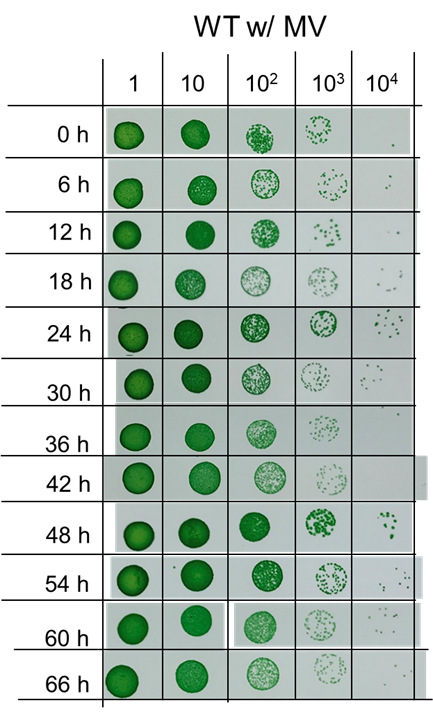


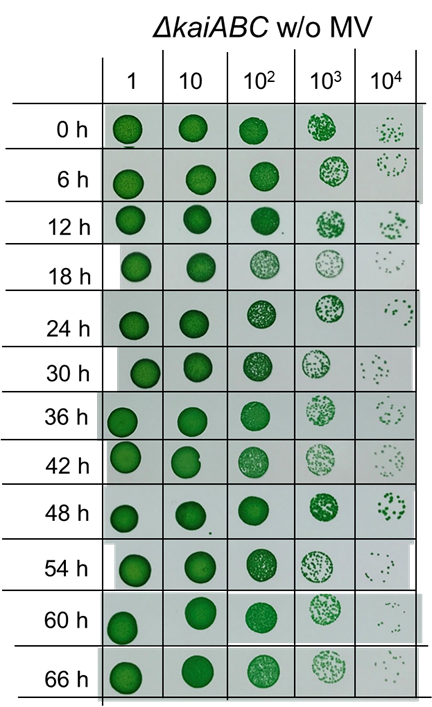

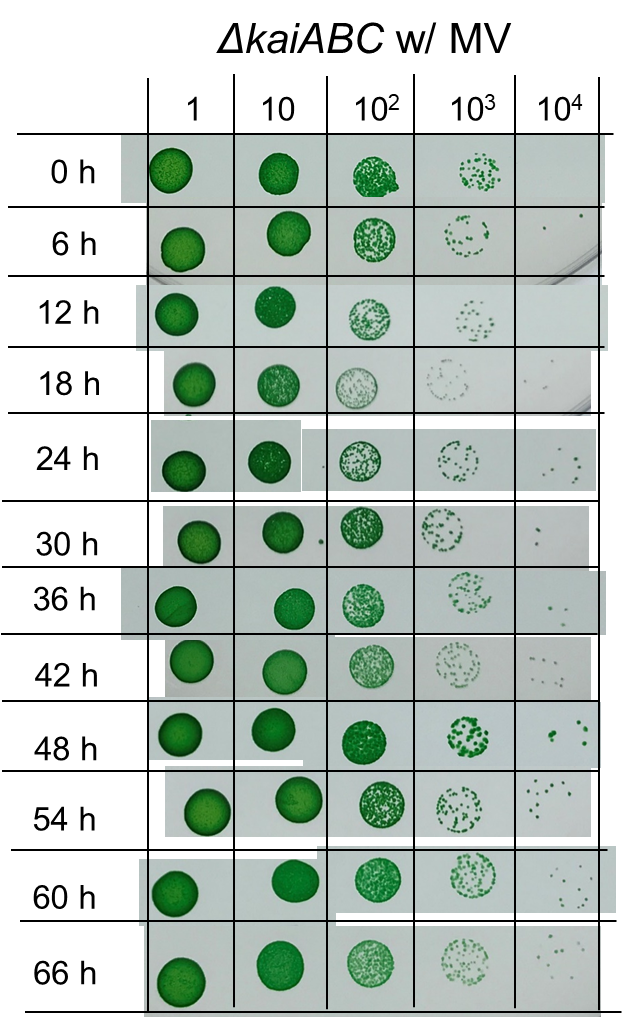


**D**

**C**

**Figure S6.** Representative photographs of colonies of samples in Fig. 4A are shown. (A) WT cells treated without MV, (B) WT cells treated with MV, (C) ΔkaiABC cells treated without MV, and (D) ΔkaiABC cells treated with MV. Numbers above and left-side on each photograph indicate dilution rate of cell suspension spotted on the agar plates, and circadian time when the MV/light treatment was performed, respectively.

**A**


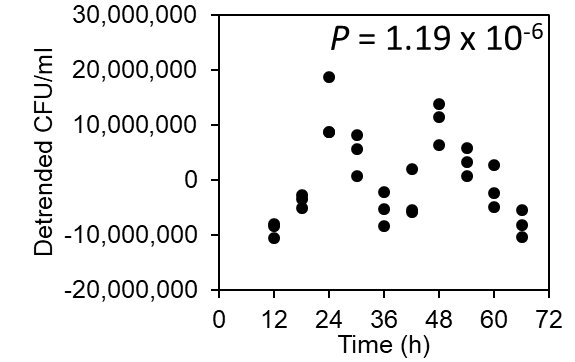


**B**


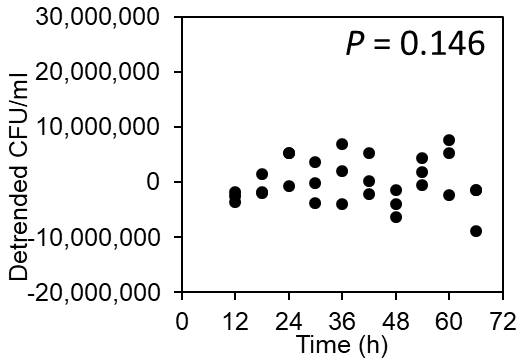


**Figure S7.** De-trended data of CFU time series of Fig 4A (See the Statistical analysis in Material and methods). (A) WT and (B) ΔkaiABC cells treated by MV, respectively. One-way ANOVA (effect of time) was performed, and *P* values are indicated.

**A**


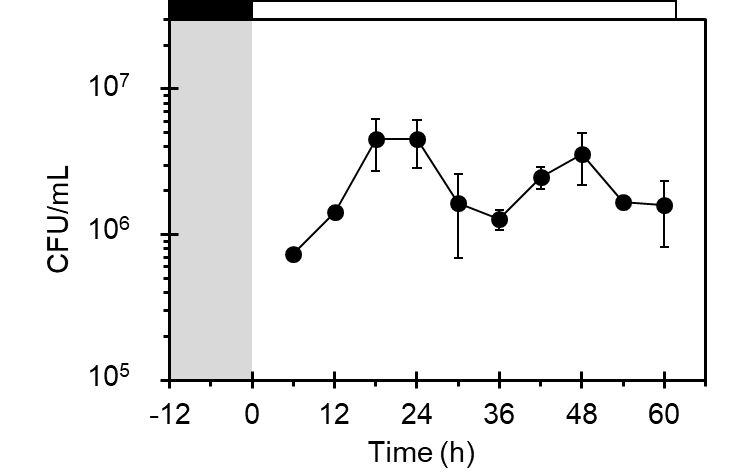


**B**


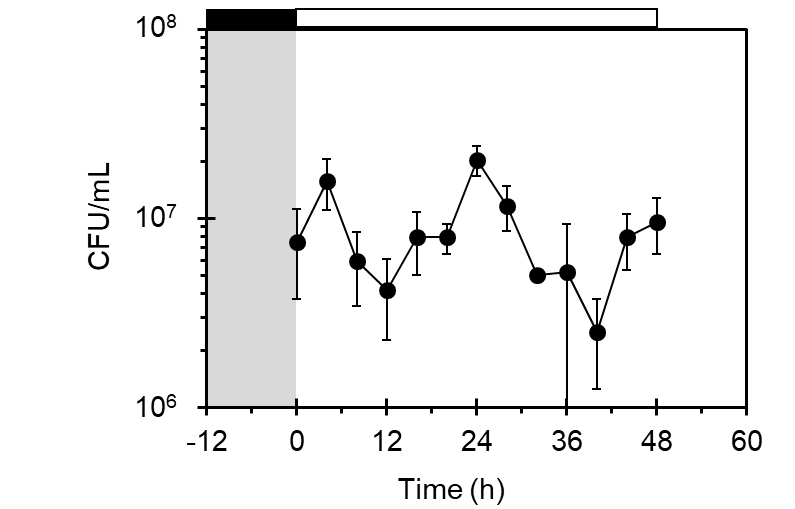


**Figure S8.** Time series of ROS-stress tolerance of cells grown in continuous light condition. Circadian rhythm of ROS-tolerance tested with WT cell culture independent from that of Fig. 4. Values are means ± SD (bars) results from three technical replicates.
